# Supplementary material for: A Potent Inhibitor of the Cystic Fibrosis Transmembrane Conductance Regulator Blocks Disease and Morbidity Due to Toxigenic Vibrio cholerae
Source: Toxins (Basel). 2022 Mar 18;14(3):225. doi: 10.3390/toxins14030225 (PMC8948642; doi:10.3390/toxins14030225)
Supplement: Supplementary file 1 [file toxins-14-00225-s001.zip › toxins-1621671-supplementary.pdf]

toxins

MDPI

Supplementary Materials

A Potent Inhibitor of the Cystic Fibrosis Transmembrane Conductance Regulator Blocks Disease and Morbidity Due to Toxigenic *Vibrio cholerae*

Fabian Rivera-Chávez, Bradley T. Meader, Sinan Akosman, Vuk Koprivica, and John J. Mekalanos

Table S1. *V. cholerae* strains used in this study.

Strain	Genotype	Reference
V. cholerae	El Tor biotype (Sm^r) C6706	Lab Collection
V. cholerae	Classical O1 O395	Lab Collection
V. cholerae	Haiti-1 (H1)	[11,30]
